# Canada’s First National Oral Health Research Strategy (2024–2030)

**DOI:** 10.1177/00220345241299360

**Published:** 2024-12-19

**Authors:** L.D. Rock, G. Akade, H. Al-Waeli, S. Allin, K. Altabtbaei, N. Ameli, C. Bassim, C. Bedos, P. Benbow, A.Y. Bhagirath, D. Chvartszaid, V. D’Souza, K. Da Silva, A. Elseoudi, A. Fadl, B. Ganss, P. Ghanouni, S. Ghavami, N. Gomaa, S. G. Gong, D. Graf, G.D. Guttmann, A. Jessani, P. Kengne Talla, L. Kenwell, M. Khurram, A. Leask, S. Madathil, S. McKinstry, H. Mulhall, B. Nicolau, O.O. Olatosi, C. Powder, F. Propser, C. Quiñonez, S. Abbasgholizadah Rahimi, D.P. Richards, M. Rouabhia, R.J. Schroth, P. Sharma, H. Szabo-Rogers, A. Velly, L. A.C. Vieira, D.T. Wu, K. Zhou, P.J. Allison

**Affiliations:** 1 Faculty of Dentistry, Dalhousie University, Halifax, Canada; 2 Faculty of Dental Medicine and Oral Health Sciences, McGill University, Montreal, Canada; 3 Institute of Health Policy, Management and Evaluation, University of Toronto, Toronto, Canada; 4 School of Dentistry, University of Alberta, Edmonton, Canada; 5 Faculty of Health Sciences, McMaster University, Hamilton, Canada; 6 School of Health Studies, Algonquin College, Ottawa, Canada; 7 Faculty of Dentistry, University of Toronto, Toronto, Canada; 8 Schulich School of Medicine and Dentistry, University of Western Ontario, London, Canada; 9 Department of Biochemistry and Molecular Medicine, Faculty of Medicine, University of Montreal, Canada; 10 College of Dentistry, University of Saskatchewan, Saskatoon, Canada; 11 School of Occupational Therapy, Dalhousie University, Halifax, Canada; 12 Department of Anatomy and Cell Science, University of Manitoba. Winnipeg, Canada; 13 Faculty of Dentistry, University of British Columbia, Vancouver, Canada; 14 Woody L. Hunt School of Dental Medicine, Texas Tech University Health Sciences Centre, El Paso, TX, USA; 15 Indigenous Dental Association of Canada, Saskatoon, Canada; 16 Canadian Institutes of Health Research, Institute of Musculoskeletal Health and Arthritis, University of British Columbia, Vancouver, BC, Canada; 17 Dr. Gerald Niznick College of Dentistry, University of Manitoba. Winnipeg, Canada; 18 Canadian Dental Therapists Association, Regina, Canada; 19 First Nations Inuit Health Branch (Atlantic), Indigenous Services Canada, Halifax, Canada; 20 Department of Family Medicine, University of Montreal, Montreal, Canada; 21 Faculty of Dentistry, Université Laval, Québec, Canada; 22 Chronic Disease and Injury Prevention, Regin of Peel Public Health, Mississauga, Canada; 23 Department of Anatomy, Physiology and Pharmacology, College of Medicine, University of Saskatchewan, Saskatoon, Canada; 24 Harvard School of Dental Medicine, Harvard University, Boston, MA, USA

**Keywords:** health services research, health inequalities, oral-systemic disease(s), public health, publishing, access to care

## Abstract

Recent years have seen significant positive changes and developments in oral health–related policy and data on oral health and oral health care in Canada. Simultaneously, on the international stage, the momentum for oral health and related research continues to build. These changes have led to an initiative to create Canada’s first National Oral Health Research Strategy (NOHRS), which was recently published by the Canadian Institutes of Health Research–Institute of Musculoskeletal Health and Arthritis (Allison and Rock 2024). In this communication, we describe the process that was used to undertake this work. We present the resulting guiding principles, the research priority areas, and the framework that emerged, which included 6 strategic priorities grouped into 3 themes: (A) Leading Issues: (1) access to care, (2) inequities, identities, and oral health; (B) Emerging Methods: (3) artificial intelligence, (4) omics; and (C) Overarching Approaches: (5) environmental sustainability, (6) knowledge mobilization and implementation science. In addition, NOHRS includes a series of proposed goals and a timeline over the coming years. The point is to encourage a broad range of individuals and groups of people to engage with this high-level strategy and create plans to implement it. This strategy directly answers the call by the World Health Organization for countries to establish a national oral health research strategy (World Health Organization 2024). We have engaged in an extensive, broad consultative process, resulting in a Canadian NOHRS that is tailored to the needs of our community. Its aim is to galvanize our community into action to address the priorities we have identified. By engaging in this process, we build upon multiple oral health–related initiatives in Canada and on the international stage. We hope to inspire and facilitate similar, much-needed work elsewhere.

## Introduction

After many years of oral health–related policy stagnation and limited national data on oral health and oral health care in Canada, recent years have seen significant changes and developments in both fields. National surveys of the oral health and oral health care of people living in Canada were last performed approximately 15 years ago (Government of Canada 2010; First Nations Information Governance Centre 2011; Health Canada 2011) and before that in the 1970s (Nutrition Canada 1977). During this period of the 1970s to the early 2010s, Canada’s privately dominated dental care delivery system expanded to provide approximately 94% of all dental care. The remaining 6% was an ad hoc collection of small public programs run by Canada’s federal, provincial, and territorial governments, targeting a range of marginalized groups struggling to obtain dental care, such as Indigenous populations, young children, and seniors (Quiñonez 2021).

This ad hoc, “laissez-faire” approach to dental care in Canada resulted in increasing numbers of people in Canada having difficulties accessing dental care. In 2014, the Canadian Academy of Health Sciences reported the multiple inequities in accessing dental care that a wide range of marginalized Canadian groups were experiencing (Canadian Academy of Health Sciences 2014), while another report documented that dental caries was the most common reason 1- to 5-year-old children in Canada needed day-care general anesthesia (Schroth et al. 2016). A more recent report highlighted that an increasing proportion of Canadians had no dental insurance and were avoiding consulting a dentist due to cost (Statistics Canada 2023). This, in turn, has resulted in increasing numbers of Canadians trying to obtain dental care through hospital emergency departments (Quiñonez et al. 2009, 2011). These scientific reports were mirrored by increasing media reports of the problems many Canadians were having accessing dental care, to the point where provincial and federal political parties began to discuss the subject in electoral campaigns (Woodend 2019). This culminated in the current federal government launching a Canadian Dental Care Plan (CDCP) for a substantial number of people living in Canada, with those families earning less than $70,000 CAD receiving full coverage of their dental care and those earning between $70,000 and $90,000 CAD having partial coverage (Government of Canada 2023).

During this same period, the relatively small oral health–related research community in Canada also struggled to obtain funding and build capacity. The primary source of health research funding in Canada is through the Canadian Institutes of Health Research (CIHR), which comprises 13 institutes, including the Institute of Musculoskeletal Health and Arthritis (IMHA), tasked with supporting oral health research and other domains (Canadian Institutes of Health Research 2024). Data for funding in open competitions at CIHR showed that oral health–related research projects and funding for members of Canada’s 10 faculties of dentistry were very low. In 2015, 2019, and 2023, CIHR-IMHA funded the Network for Canadian Oral Health Research (NCOHR). CIHR also invested $3.3 M to support the oral health data collection element of the Canadian Health Measures Survey (CHMS), cycle 7 (Canadian Institutes of Health Research 2023b). This ongoing survey will provide data on the oral and general health of independent-living individuals aged 1 to 79 years in Canada, enabling comparison with the cycle 1 CHMS data collected in 2007 to 2009 (Government of Canada 2010).

In parallel to these Canadian initiatives, momentum for oral health and related research began to build internationally. In the United Kingdom, the National Institute for Health and Care Research agreed on a list of oral and dental research priorities (National Institute for Health Care Research 2018), and Iran published a needs assessment an research priorities in oral and dental health (Rafie et al. 2019). *The Lancet* published a series on oral health (Peres et al. 2019; Watt et al. 2019) in 2019 and established a Lancet Commission on oral health in 2020, the US National Institute of Dental and Craniofacial Research 2021–2026 launched their strategic plan (National Institutes of Health National Institute of Dental and Craniofacial Research 2022), and Peru published national priorities for oral health research (Echevarria-Goche et al. 2023). At the same time, the World Health Organization (WHO) consulted extensively and then published the Global Strategy and Action Plan on Oral Health (GSAPOH) 2023–2030 (World Health Organization 2024). Objective 6 of that strategy asserts that “by 2030, 50% of countries have a national oral health research agenda fused on public health and population-based interventions.”

These transformative changes in the political and dental care landscape, along with the growing momentum for change in oral health both nationally and internationally, led to the inception of Canada’s inaugural National Oral Health Research Strategy (NOHRS) (Allison and Rock 2024).

## Goals

The goals of Canada’s NOHRS are to (1) galvanize the oral health and broader community to collaborate around the strategic priorities identified and (2) leverage our strengths in existing areas to foster and nurture new research fields and leaders, while establishing the necessary infrastructure required to address the identified priorities. In other words, NOHRS serves as a catalyst for change within Canada’s oral health landscape, responding to significant policy shifts, increased data collection, plus numerous international calls for transformations in oral health care policies and research.

## Process

The process was initiated through the leadership of CIHR-IMHA convening a NOHRS steering committee in the summer of 2022 comprising leaders of the Association of Canadian Faculties of Dentistry, the Canadian Association for Dental Research, and NCOHR. In the autumn of 2022, a call for expressions of interest was issued to the community to participate in the launch event. This was a 2-day meeting in Ottawa in March 2023, with the goal of generating initial ideas for the strategy. There were approximately 70 individuals representing a wide range of research and oral health care sectors as well as patient and community groups. A report summarizing the meeting was subsequently distributed and made publicly available (Canadian Institutes of Health Research 2023a). Concurrently, a broad survey was conducted, seeking feedback on the 6 initial priority areas resulting from the meeting report, and an open call was distributed requesting volunteers to be involved in drafting sections of the strategy. From this pool of volunteers, in the summer of 2023, the NOHRS steering committee identified co-chairs to lead the strategy development as well as co-chairs and writing teams for each of the 6 priorities. The so-formed priority writing teams consisted of approximately 5 or 6 members each who then invited a student to join each team. During the autumn of 2023, these teams then drafted background material and formulated research questions and key issues to be addressed for each priority area. As a part of this process, virtual consultations were held with patient and community representatives to gather their input and feedback on the writing teams’ initial ideas. Each working group produced a brief report on what they had heard from the consultation attendees and how it would affect their section of the strategy. In the meantime, the project co-chairs collaborated with the steering committee and writing team co-chairs to generate the NOHRS framework, including its rationale, goals, guiding principles, and the way forward once it had been launched. The material was integrated into a comprehensive first draft of the NOHRS document in January 2024. Following feedback from the writing teams, a second draft was prepared and translated into French. In March 2024, both language versions were widely distributed in the community, accompanied by a survey for additional feedback. Simultaneously, select patient partners and international reviewers were engaged for their perspective, and several webinars were held to explain the draft document and solicit further input. In April 2024, the project co-chairs compiled all the feedback and produced the final document, which was formally announced and presented at the Canadian Oral Health Summit at Dalhousie University in Halifax, Nova Scotia, in June 2024 (Allison and Rock 2024).

## Result: Guiding Principles, Priorities, and Framework

**Figure 1.**
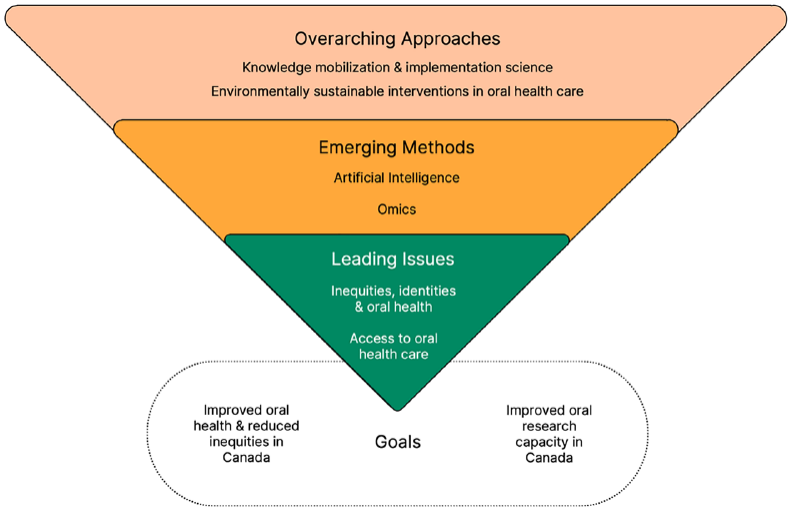
National Oral Health Research Strategy Framework. A broad consultation process generated 6 priority areas. These comprise *leading issues*, which are core problems requiring research to address them; *emerging methods* where we want to concentrate building skills, capacity, and infrastructure; and *overarching approaches*, which are both topics for research and themes applied to all the work we will engage in related to this strategy.

This broad consultation process generated 6 priority areas that were integrated into the framework demonstrated in Figure 1. The priorities comprised *leading issues* (core problems requiring research to address them), *emerging methods* (areas of focus for building skills, capacity, and infrastructure), and *overarching approaches* (topics for research and themes applied to across the strategy).

The 2 leading issues are (1) access to oral health care and (2) inequities, identities, and oral health. The former is a major problem in Canada and indeed one of the main reasons behind the recent announcement of the CDCP. Nevertheless, there remains a need for research, development, and implementation to improve access to oral health care for many marginalized groups in Canada. The second leading issue recognizes that, beyond access, there are a wide array of biological, social, commercial, and other factors that contribute to oral and general health inequity in Canada. This priority focuses on better understanding how biological and environmental factors, including people’s identities, intersect to determine their oral health status.

The 2 emerging methods are (1) artificial intelligence (AI) and oral health and (2) omics and oral health. While these methods have been used in research for some time, the NOHRS development process highlighted the need for the Canadian oral research community to strengthen its skills and infrastructure to fully leverage these tools for use in oral health research in Canada. Thus, AI and omics are considered core, evolving elements of the strategy.

The 2 overarching approaches that emerged were (1) knowledge mobilization and implementation science and (2) environmentally sustainable interventions in oral health care. The former was recognized as so crucial in our NOHRS development process in ensuring research findings are mobilized and implemented that it warranted its own priority focus, rather than serving as a mere guiding principle for the other priority topics. The development of the CDCP exemplifies the potential and necessity for the Canadian oral health research community to actively engage with diverse community groups, decision makers, health professionals, and end users to ensure research findings inform improved oral health care and policies. The second overarching approach, environmentally sustainable approaches to research, was identified due to a collective desire to prioritize sustainability in research, health care, policies, and all related developmental work. Our goal is to encourage the integration of environmentally sustainable practices by oral health research and oral health care communities in Canada.

Finally, in addition to identifying these 6 priority areas that comprise our NOHRS framework, the strategy development team agreed on a set of guiding principles to be applied to all the work related to the strategy. The guiding principles are centered around *people*, *ideas*, and *science*. In the *people* category, our guiding principles are *equity, diversity, inclusion, and accessibility; patient and community engagement*; and *capacity building*. The goal of these principles is to foster the inclusion of a wide range of individuals in NOHRS, thereby creating a larger and broader pool of people with skills to help us advance our work. In the *ideas* category, our guiding principles are *oral health is health*, *health promotion and disease prevention*, and *environmental sustainability*. With these ideas we want to integrate the concept that oral health is an integral part of overall health, moving beyond simply investigating the relationship between the two. In addition, we want to ensure that health promotion, disease prevention, and environmental sustainability are integral to all aspects of oral health research and practice. Finally, in the *science* category of guiding principles, we want to promote the concept of research and science being open to all, emphasizing collaboration and integration of individuals with diverse life experiences, including those with formal and informal training as researchers, health care providers, patients, and community members. These principles also advocate for science-informed decision-making at all levels, from research to health care to policy implementation.

## The Way Forward

**Figure 2.**
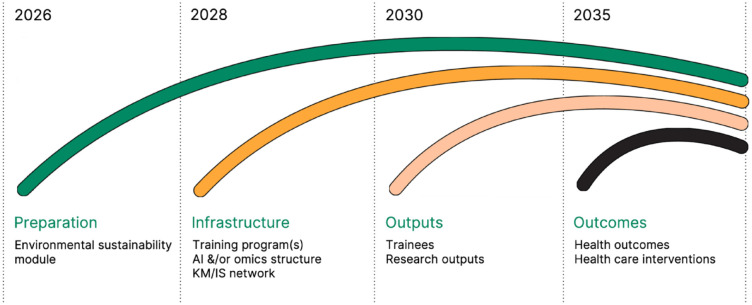
Canada’s National Oral Health Research Strategy includes a series of proposed goals and a timeline over the coming years. The goals and timeline are broad and flexible to enable those who engage and take the lead to take the ideas in the directions they believe most appropriate. These objectives integrate the need to create preparatory infrastructure (e.g., online training modules in certain fields such as environmental sustainability in research and health care), followed by training programs in relevant fields (e.g., AI and/or implementation science) and research infrastructure (e.g., in omics methodologies), before we can expect the outputs of those programs and networks, and finally, outcomes in terms of advances in improved health care and reduced inequities in health, which are our ultimate goals of this strategy.

Finally, the NOHRS includes a series of proposed goals and a timeline over the coming years (Fig. 2). The intent is to encourage engagement of this high-level strategy with a broad range of individuals and groups. The goals and timeline presented in NOHRS are intentionally rendered broad and flexible to enable those who engage to take the lead to drive forward ideas in the directions they believe most appropriate. This also recognizes that members of the community need to take ownership of the strategy and create a range of initiatives. Furthermore, this flexibility acknowledges that different funding, partnership, and support collaborations may emerge at different times and from various agencies and organizations. Finally, while it would be ideal if we were able to advance all 6 priority areas in the desired directions, creating training programs, research infrastructure, and knowledge mobilization and implementation networks that apply to all elements of the strategy, in a world of finite resources, this is unlikely. Nevertheless, the goals and timeline incorporate the need to create preparatory infrastructure (e.g., online training modules in certain fields such as environmental sustainability in research and health care), followed by training programs in relevant fields (e.g., AI and/or implementation science) and research infrastructure (e.g., in omics methodologies). This will pave the way for desired outputs of those programs and networks (e.g., in terms of learners with more expertise and experience and/or research outputs such as papers and presentations) and, finally, outcomes in terms of advances in science and knowledge in certain fields, improved health care, and reduced inequities in health, which are our ultimate goals of this strategy.

## Canada’s Strategy Compared with Those of Other Countries

In this communication, we present Canada’s NOHRS. The process and outcomes show similarities and differences compared with other countries. The United Kingdom used the James Lind Alliance’s structured approach to develop a list of 10 oral health–related research questions, whereas Iran and Peru used qualitative methods, including document reviews, semistructured interviews, questionnaires, and participatory methodologies. Our process closely resembled that of the National Institute of Dental and Craniofacial Research, involving broad consultative input from various groups and multiple opportunities for community review. Despite differing approaches, cultures, and national contexts, most strategies highlighted access to care and the integration of oral and general health as priorities. Notably, precision medicine approaches and AI were mentioned only in the Canadian and U.S. strategies, possibly because of their more recent development. Canada’s NOHRS was unique in explicitly prioritizing environmental sustainability in its research agenda.

## Conclusions and Implications

This national oral health research strategy directly answers the call by the WHO’s GSAPOH for countries to establish a national oral health research strategy (World Health Organization 2024). We have engaged in an extensive consultative process over a period of nearly 2 years, resulting in a Canadian NOHRS that is tailored to the needs of our community. Its aim is to galvanize our community into action to address the priorities we have identified and to build on our strengths. In a few years’ time, we envision having an expansive range of individuals and experts, with appropriate skills plus research data, infrastructure, and networks that can help us improve health care and reduce health inequities in Canada, while using environmentally sustainable approaches. By engaging in this oral health research process, we build on multiple oral health–related initiatives in Canada and on the international stage. Global health research is in the national interest, but answers are often not found within any single country. The announced research priorities will require international collaborative and comparative studies. We hope to inspire and facilitate similar, much-needed work elsewhere. After many years of inaction, oral health disease and oral health care are finally being recognized by many as significant problem areas requiring concerted research, development, and policy action to begin addressing them. NOHRS aims to be a driving force in this new movement.

## Author Contributions

L.D. Rock, contributed to conception and design, data acquisition, and interpretation, drafted and critically revised the manuscript; G. Akade, H. Al-Waeli, S. Allin, K. Altabtbaei, N. Ameli, C. Bassim, C. Bedos, P. Benbow, A.Y. Bhagirath, D. Chvartszaid, V. D’Souza, K. Da Silva, A. Elseoudi, A. Fadl, B. Ganss, P. Ghanouni, S. Ghavami, G.D. Guttmann, A. Jessani, P. Kengne Talla, L. Kenwell, M. Khurram, S. Madathil, B. Nicolau, O. Olatosi, C. Powder, F. Propser, S. Abbasgholizadah Rahimi, H. Szabo-Rogers, A. Velly, L.A.C. Vieira, D. Wu, K. Zhou, contributed to acquisition and interpretation, critically revised the manuscript; N. Gomaa, S.G. Gong, D. Graf, A. Leask, S. McKinstry, C. Quiñonez, M. Rouabhia, R.J. Schroth, P. Sharma, contributed to acquisition and interpretation, drafted and critically revised the manuscript; H. Mulhall, contributed to conception and design, critically revised the manuscript; D.P. Richards, contributed to conception and design, critically revised the manuscript; P.J. Allison, contributed to conception and design, data acquisition and interpretation, drafted and critically revised the manuscript. All authors gave their final approval and agree to be accountable for all aspects of the work.
